# Efficient design and analysis of randomized controlled trials in rare neurological diseases: An example in Guillain-Barré syndrome

**DOI:** 10.1371/journal.pone.0211404

**Published:** 2019-02-20

**Authors:** Nikki van Leeuwen, Christa Walgaard, Pieter A. van Doorn, Bart C. Jacobs, Ewout W. Steyerberg, Hester F. Lingsma

**Affiliations:** 1 Centre for Medical Decision Making, Department of Public Health, Erasmus University Medical Center, Rotterdam, The Netherlands; 2 Department of Neurology, Erasmus University Medical Center, Rotterdam, The Netherlands; 3 Department of Immunology, Erasmus University Medical Center, Rotterdam, The Netherlands; 4 Department of Medical Statistics and Bioinformatics, Leiden University Medical Centre, Leiden, The Netherlands; National Yang-Ming University, TAIWAN

## Abstract

**Background:**

Randomized controlled trials (RCTs) pose specific challenges in rare and heterogeneous neurological diseases due to the small numbers of patients and heterogeneity in disease course. Two analytical approaches have been proposed to optimally handle these issues in RCTs: covariate adjustment and ordinal analysis. We investigated the potential gain in efficiency of these approaches in rare and heterogeneous neurological diseases, using Guillain-Barré syndrome (GBS) as an example.

**Methods:**

We analyzed two published GBS trials with primary outcome ‘at least one grade improvement’ on the GBS disability scale. We estimated the treatment effect using logistic regression models with and without adjustment for prognostic factors. The difference between the unadjusted and adjusted estimates was disentangled in imbalance (random differences in baseline covariates between treatment arms) and stratification (change of the estimate due to covariate adjustment). Second, we applied proportional odds regression, which exploits the ordinal nature of the GBS disability score. The standard error of the estimated treatment effect indicated the statistical efficiency.

**Results:**

Both trials were slightly imbalanced with respect to baseline characteristics, which was corrected in the adjusted analysis. Covariate adjustment increased the estimated treatment effect in the two trials by 8% and 18% respectively. Proportional odds analysis resulted in lower standard errors indicating more statistical power.

**Conclusion:**

Covariate adjustment and proportional odds analysis most efficiently use the available data and ensure balance between the treatment arms to obtain reliable and valid treatment effect estimates. These approaches merit application in future trials in rare and heterogeneous neurological diseases like GBS.

## Introduction

RCTs are the standard to investigate the effectiveness of medical interventions. However, RCTs are challenging in rare heterogeneous diseases. The randomization process in RCTs ensures that observed and unobserved patient characteristics on average are similar between treatment arms[1]. However, it does not ensure full balance[1]. Different baseline risks for outcome can arise between treatment arms, simply due to chance[1]. In diseases with large between-patient differences in natural disease course, severity and outcome, small imbalances in covariates between the treatment arms may, positively or negatively, affect the estimated treatment effect.

Sample sizes in RCTs in rare diseases are usually small. Small trials are a subject to a greater chance of imbalance than large trials[1]. Moreover, small RCTs can easily fail to detect treatment benefits, due to lack of statistical power. In rare neurological disorders, such as inflammatory neuropathies like Guillain-Barré syndrome (GBS), Chronic Inflammatory Demyelinating Polyneuropathy (CIDP) and Multifocal Motor Neuropathy (MMN), this heterogeneity and rarity is a major challenge for conducting RCTs.

Two approaches to optimize RCT design and analysis that have been successfully applied in other acute neurological diseases such as stroke and traumatic brain injury are covariate adjustment and ordinal analysis[2–4]. (Table 1) Covariate adjustment is a statistical method that adjusts the treatment effect for baseline risk on poor outcome in the treatment arms. When the treatment arms are imbalanced, an unadjusted analysis is suboptimal to estimate the treatment effect. In addition, previous studies found that covariate adjustment could increase statistical power[1, 5–9]. Ordinal analysis is an approach to analyze a full ordinal outcome scale instead of a dichotomized version. Although these techniques already have been successfully applied in stroke and traumatic brain injury, it is still relevant to study this in other diseases like GBS, since the effect of the different approaches can work out differently in different study settings. The most commonly used outcome in GBS is the ordinal GBS disability score, consisting of seven categories. Usually the scale is dichotomized into favorable or unfavorable outcome, or the improvement on the GBS disability score from admission calculated and dichotomized as minimal one grade improvement. In ordinal analysis the outcome is not dichotomized but analyzed as the full ordinal scale with proportional odds analysis, preventing loss of information[10]. Simulation studies and empirical validation studies in other fields have demonstrated that proportional odds analysis increases statistical power in RCTs[10–13].

**Table 1:** Distribution of baseline predictors and outcome distribution in two randomized controlled trials in GBS.

	PE vs IVIg trial			IVIg + placebo vs IVIg + Methylprednisolon (IVIg vs MP) trial		
	Total (n = 146)	Control (PE) (n = 73)	Treatment (IVIg) (n = 73)	Total (n = 221)	Control (IVIg) (n = 111)	Treatment (IVIg+MP) (n = 110)
**Age** (Median, Interquartile Range 25^th^-75^th^ Percentile)	49 (32–63)	51 (33–66)	47 (32–61)	55 (35–67)	52 (35–67)	57 (34–68)
**Preceding diarrhea**	27 (19%)	16 (22%)	11 (15%)	60 (27%)	30 (27%)	30 (27%)
**GBS disability score at admission**						
Able to walk over 10m open space with help	29 (20%)	16 (22%)	13 (18%)	58 (26%)	32 (30%)	26 (24%)
Bedridden or chair bound	92 (63%)	44 (60%)	48 (66%)	53 (49%)	78 (70%)	75 (68%)
Needs ventilation for at least a part of the day	25 (17%)	13 (18%)	12 (16%)	10 (5%)	1 (1%)	9 (8%)
**Predicted probability of one or more grades** **improvement on the GBS disability score after 4 weeks**	0.43	0.41	0.45	0.62	0.64	0.60
**One or more grades improvement on the GBS disability score after 4 weeks**	63 (43%)	25 (34%)	38 (52%)	137 (62%)	63 (57%)	74 (67%)
**GBS disability score after 4 weeks**						
0 = Healthy	0 (0%)	0 (0%)	0 (0%)	5 (2%)	0 (0%)	5 (5%)
1 = Minor symptoms	16 (11%)	6 (8%)	10 (14%)	37 (17%)	24 (22%)	13 (12%)
2 = Able to walk 10m unassisted but not able to run	30 (21%)	12 (16%)	18 (25%)	74 (34%)	31 (28%)	43 (39%)
3 = Able to walk over 10m open space with help	19 (13%)	9 (12%)	10 (14%)	22 (10%)	10 (9%)	12 (11%)
4 = Bedridden or chair bound	48 (33%)	27 (37%)	21 (29%)	54 (24%)	31 (28%)	23 (21%)
5 = Needs ventilation for at least a part of the day	31 (21%)	17 (23%)	14 (19%)	26 (12%)	14 (13%)	12 (11%)
6 = Dead	2 (1%)	2 (3%)	0 (0%)	3 (1%)	1 (1%)	2 (2%)

To test the applicability and value of these approaches in rare and heterogeneous neurological diseases, we use Guillain-Barré syndrome (GBS) as an example. GBS is a life-threatening acute immune-mediated polyradiculoneuropathy[14, 15], which requires early diagnosis and hospital admission for accurate monitoring, treatment and supportive care. Some patients may show spontaneous and full recovery, while others require ventilation at an ICU for months and remain severely disabled. Several RCTs have successfully been conducted in GBS[16–18].

We aimed to explore the potential benefit of covariate adjustment and proportional odds analysis in rare and heterogeneous neurological diseases, compared to the conventional statistical approaches. We hereto re-analyzed two RCTs in GBS.

## Methods

### Patient population

We analyzed data from two RCTs in GBS, the Plasma Exchange (PE) vs Intravenous Immunoglobulin (IVIg) (PE vs IVIg) trial[17] and the IVIg and placebo versus IVIg and Methyl-Prednisolone (MP) (IVIg vs MP) trial[18], conducted between 1986 and 2000. In the PE vs IVIg trial, the control group received IVIg and the treatment group received PE. In the IVIg vs MP trial, the patients receiving IVIg and placebo were considered as control patients and the patients receiving IVIg and MP were considered as treated patients. The primary outcome in both trials was improvement (corresponding to lower GBS disability scores) by one or more grades on the GBS disability score after 4 weeks. The GBS disability score is an ordinal scale ranging from 0 = healthy to 6 = dead. However, in order to estimate treatment effects for a positive outcome for all the analyses, we used the reversed GBS disability score at 4 weeks, to keep the estimates easy to compare. For all the regression models used in this paper, higher numbers (in outcome) mean better health outcomes.

### Statistical analysis

The predicted probabilities for one grade improvement on the GBS disability score were calculated and used as a measure for baseline risk to indicate potential unbalance between the treatment arms in baseline characteristics.

To estimate treatment effects, we used two commonly used primary (dichotomous) outcomes in GBS trials as reference; (1) favorable outcome (0–2) on the GBS disability scale at 4 weeks as outcome and (2) minimal one grade improvement on the GBS disability score between the moment of randomization and 4 weeks as outcome, both analyzed with binary logistic regression without covariate adjustment. Consequently, these references were compared with the two approaches under study: covariate adjustment and ordinal analysis.

### Covariate adjustment

With covariate adjustment, conditional treatment effects are estimated with regression models. Adjusting for GBS disability score at admission results in an estimated treatment effect for a patient with a given GBS disability score, while unadjusted analysis results in an average estimated treatment effect over all patients, irrespective of the GBS disability score. Unadjusted analysis is expressed by the following formula:

logodds(improvement)=α+β*treatment,

where improvement is by one or more grades on the GBS disability score, and treatment is an indicator for the randomization arm. The coefficients *α* and *β* indicate the intercept and regression coefficient for treatment. In logistic regression, exp(*β*) indicates the odds ratio (OR).

For adjusted analysis, we used three well-known predictors of outcome[19, 20]: age, preceding diarrhea and GBS disability score at admission. The covariate adjusted model is expressed by the following formula:

logodds(improvement)=α+β*treatment+β1*age+β2*precedingdiarrhea+β3*GBSdisabilityscoreatadmission

This results in an adjusted regression coefficient *β* for the estimated treatment effect. In the trial analysis, the observed difference of the unadjusted and adjusted regression coefficient for the treatment variable is a result of imbalance and stratification[8]. We hereto calculated the linear predictor based on age, diarrhea and GBS disability score at admission. We then calculated the difference in treatment effect that was attributable to imbalance as the difference between the mean value of the linear predictor between the treatment arms[8]. The remaining part of the difference between the unadjusted and the adjusted treatment effect was attributed to stratified estimation, i.e. conditioning on covariates[8].

### Proportional odds analysis

For ordinal analysis we used proportional odds logistic regression to exploit the ordinal nature of the GBS disability score. A proportional odds logistic regression model was fitted with the GBS disability score collapsed to a 5-point scale. We combined both healthy (0) and minor symptoms (1), as well as needs ventilation at least a part of the day (5) and dead (6) because of small numbers in these extreme categories. We used the reversed GBS disability scale to estimate treatment effects on a positive outcome, and to keep these estimates comparable to the estimates of the other logistic regression models on positive dichotomous outcomes (improvement and favorable outcome). The proportional odds model uses an ordinal outcome variable with more than two possible categories. It estimates a common OR over all possible cut-offs of the outcome scale. Next, we used the difference between the GBS disability score at admission and the GBS disability score at four weeks as outcome. A proportional odds logistic regression model was used to analyse the difference in GBS disability score.

### Treatment effect estimates

The coefficient *β* of the treatment effect and the corresponding standard error (SE) were calculated for the four approaches to analyse outcome, with and without covariate adjustment. The SE of the treatment effect indicates the precision of the calculated treatment effect. The SEs in the proportional odds regression models are expected to be smaller than those in the logistic models. Both trials were analysed with complete case analysis, ignoring 1 and 4 patients with incomplete baseline data. Statistical analyses were performed in R Statistical Software version 2.15.3 using the *rms* package (R Foundation for Statistical Computation, Vienna, Austria).

## Results

### Patient population and reference strategies

We analysed data from 146 patients in the PE vs IVIg trial and 221 patients in the IVIg vs IVIg+MP trial. Both trials were slightly imbalanced with regard to the baseline characteristics. In the IVIg vs IVIg+MP trial the treatment group (with MP) had a probability of 0.60 to improve at least one grade on the GBS disability score compared to a predicted probability of 0.64 in the control group (without MP). So without any treatment, the prognosis of the treatment arm was slightly better. An opposite distribution of baseline covariates between treatment arms is shown in the PE vs IVIg trial. The treatment group (PE) has a higher predicted probability (0.45) to improve at least one grade on the GBS disability score compared to the control group (IVIg; predicted probability 0.41, Table 1).

Regarding the actual outcome, 63 (57%) control patients treated with IVIg and placebo and 74 (67%) patients treated with IVIg and methylprednisolone improved minimal one grade on the GBS disability score after 4 weeks. In the other trial, 25 (34%) control patients treated with IVIg and 38 (52%) patients receiving PE improved minimal one grade on the GBS disability score after 4 weeks (S1 Appendix).

The treatment under study in both trials had a positive effect on health outcomes. With the reference strategy of logistic regression on a favorable GBS disability scale (0–2) at 4 weeks as outcome, the estimated treatment OR was 1.80 (95% confidence interval (CI) 0.84–3.85, SE 0.39, p = 0.13) in the PE vs IVIg trial and 1.69 (95% CI 0.93–3.08, SE 0.31, p = 0.09) in the IVIg vs IVIg+MP trial. The treatment effect estimates on one grade improvement were slightly larger (Table 2).

**Table 2:** Treatment effect analysis: Unadjusted and adjusted binary and proportional odds logistic regression.

		PE vs IVIg trial (n = 146) Unadjusted	Adjusted*	IVIg + placebo vs IVIg + Methylprednisolon (IVIg vs MP) trial (n = 221) Unadjusted Adjusted*	
**Binary logistic regression–** **GBS disability 3–6 vs 0–2** ^§^	OR (95% CI)	1.90 (0.93–3.87)	1.80 (0.84–3.85)	1.27 (0.75–2.15)	1.69 (0.93–3.08)
	SE	0.36	0.39	0.27	0.31
	P-value	0.08	0.13	0.38	0.09
**Binary logistic regression–improvement on GBS disability score**	OR (95% CI)	2.08 (1.07–4.06)	1.95 (0.96–4.00)	1.57 (0.91–2.71)	1.96 (1.08–3.56)
	SE	0.34	0.36	0.28	0.31
	P-value	0.03	0.06	0.11	0.03
**Proportional odds logistic regression–reversed GBS disability score at 4 weeks** ^^^	OR (95% CI)	1.76 (0.98–3.19)	1.76 (0.98–3.19)	1.12 (0.70–1.80)	1.41 (0.87–2.28)
	SE	0.30	0.30	0.24	0.25
	P-value	0.06	0.06	0.63	0.17
**Proportional odds logistic regression–Δ GBS disability score (grades improvement between admission and 4 weeks)**	OR (95% CI)	1.93 (1.07–3.49)	1.80 (0.99–3.27)	1.43 (0.89–2.30)	1.34 (0.89–2.32)
	SE	0.30	0.30	0.24	0.25
	P-value	0.03	0.05	0.14	0.14

*Adjustment for age, preceding diarrhea and GBS disability score at admission.

§ 0 = Healthy / 1 = Minor symptoms / 2 = Able to walk 10m unassisted but not able to run / 3 = Able to walk over 10m open space with help / 4 = Bedridden or chair bound / 5 = Needs ventilation for at least a part of the day / 6 = Dead

^ In order to estimate the treatment effect for a positive outcome, we used the reversed GBS disability score at 4 weeks

### Covariate adjustment

With covariate adjustment, the estimated treatment effect was larger in the IVIg vs IVIg+MP trial, partly as a result of adjustment, which makes the estimates more extreme, and partly because of the imbalance at baseline. Poorer prognosis at baseline for the intervention (IVIg + MP) group implied a +31% increase in the adjusted treatment effect (Table 3). The stratification effect of adjustment was an additional 18% increase in the treatment effect (OR = 1.96). In contrast, the treatment effect was smaller with adjustment for baseline characteristics in the PE vs IVIg trial. The stratification effect increased the treatment effect with 8%, but the better prognosis in the intervention (IVIg) group at baseline reduced the estimated treatment effect by -24%. The net effect was a difference in treatment effect of -16%. These results were similar for all binary and ordinal outcome analyses (Table 2).

**Table 3:** Results of unadjusted and adjusted binary logistic regression analysis of the effect of treatment versus control on GBS disability score at four weeks in both PE vs IVIg trial (n = 146) and the IVIg + placebo vs IVIg + Methylprednisolon (IVIg vs MP) trial (n = 221).

		OR	Coefficient	Absolute difference in treatment effect between adjusted and unadjusted	Imbalance between treatment arms	Relative difference in treatment effect between adjusted and unadjusted due to imbalance	Relative difference in treatment effect between adjusted and unadjusted due to stratification
		**PE vs IVIg trial**					
**Unadjusted**		**2.08**	**0.73**				
**Adjusted for age, preceding diarrhea and GBS disability score at admission**		**1.95**	**0.67**	**- 0.06**^^^	**-0.12**	-16%*	**8%**^#^
		**IVIg vs MP trial**					
**Unadjusted**		**1.57**	**0.45**				
**Adjusted for age, preceding diarrhea and GBS disability score at admission**		**1.96**	**0.67**	**0.22**^^^	**0.14**	31%*	**18%**^#^

^ Adjusted coefficient–Unadjusted coefficient

* Imbalance between treatment arms / Unadjusted coefficient

# (Absolute difference in treatment effect between adjusted and unadjusted—Imbalance between treatment arms) / Unadjusted coefficient.

### Proportional odds analysis

**Fig 1:**
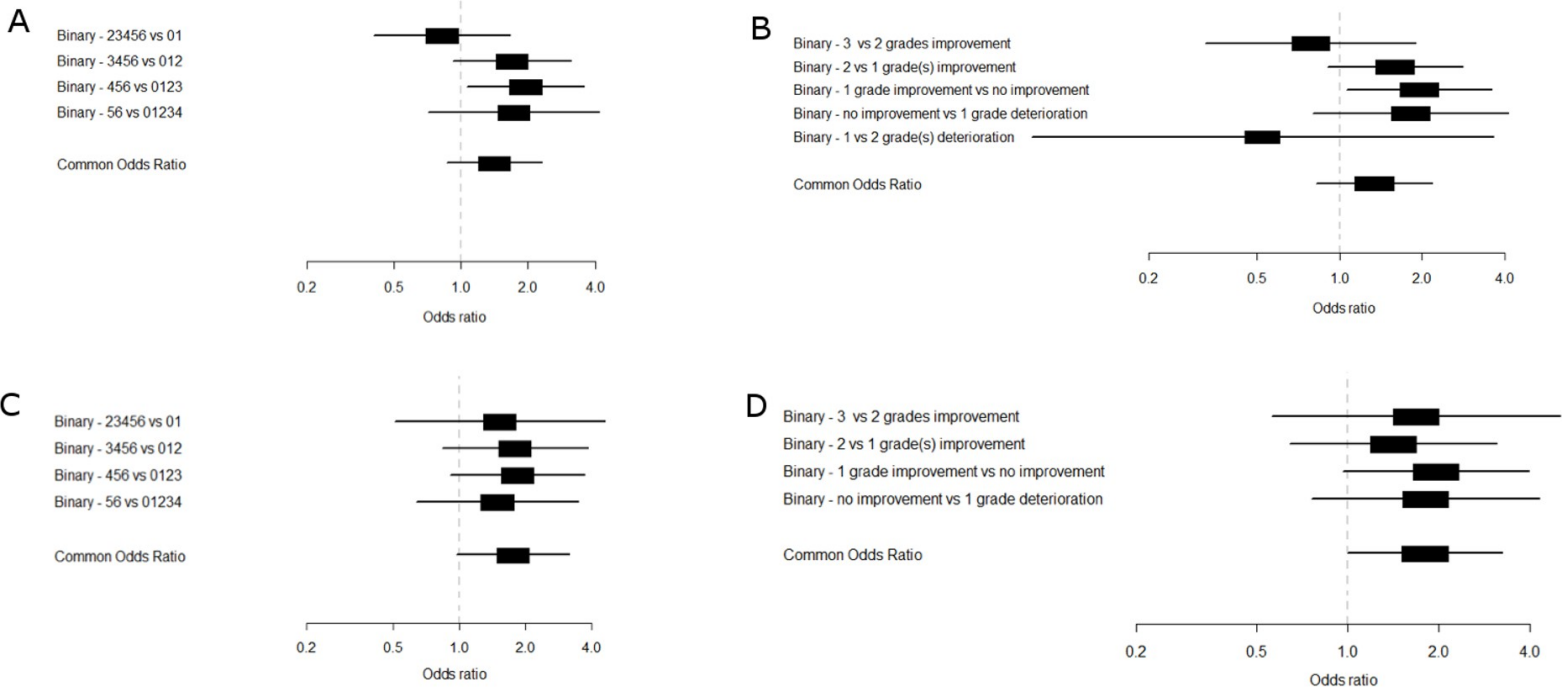
Treatment effect analysis: forest plots of the adjusted binary and proportional odds logistic regression in the IVIg + placebo vs IVIg + Methylprednisolon (IVIg vs MP) trial (a and b) and PE vs IVIg trial (c and d) show smaller confidence intervals for the common odds ratio compared to the binary estimates.

For illustration of the proportional odds analyses we calculated the treatment effect estimates (ORs) for each cut-off of the reversed ordinal scale. The common OR can be interpreted as the pooled estimate of these binary ORs. The treatment under study in both trials had a positive effect on health outcomes in all the ordinal analyses. In the PE vs IVIg trial the ORs over each cut-off were relatively similar (Fig 1C and 1D. The common OR was similar as well, but the SE and CI were smaller. In the IVIg vs IVIg+MP trial, the ORs were more variable (Fig 1A and 1B. The common OR was less extreme compared to ORs for the cut-off used in the reference approach (0–2 vs. 3–6 and minimal one grade improvement vs. no improvement). But again, the SE and CI were smaller. This can also be seen in Table 2; in all analyses, the proportional odds analysis on the GBS disability score after four weeks and on the improvement on the GBS disability score resulted in lower SEs of the treatment effect compared to the binary approaches.

## Discussion

In this study we assessed the potential benefit of the use of covariate adjustment and proportional odds analysis in RCTs compared to the conventional method, by reanalyzing two GBS trials. We found that covariate adjustment increased the estimated treatment effect in one trial, and decreased the estimated treatment effect in the other trial, due to imbalances in baseline characteristics between the treatment arms. Although such imbalances are fully due to chance if a proper randomization procedure is followed, our results illustrate that their impact on interpretability of treatment effect estimates can be substantial and can be different in several study settings. We found that the proportional odds analysis resulted in lower standard errors and thus smaller confidence intervals of the treatment effect estimate compared to the conventional method of logistic regression on dichotomized outcome measures. Thus, dichotomization of ordinal outcome measures does not merit application. In future trials in rare and heterogeneous neurological diseases like GBS both covariate adjustment and proportional odds analysis are advised.

### Covariate adjustment

On expectation, covariate adjustment leads to more extreme treatment effect estimates and larger standard errors for non-linear regression models.[21] The p values are a function of the treatment effect estimates and standard error. With covariate adjustment the increase in treatment effect estimate will outweigh increased in standard error and the p values will be lower compared to unadjusted analysis[21].

Indeed, we found increased standard errors in all adjusted analyses compared to the unadjusted analyses. The better prognosis in the treatment group decreased the treatment effect estimate *β* after covariate adjustment in the PE vs IVIg trial. In the IVIg vs MP trial, the treatment group had a lower probability of favorable outcome. Therefore, in the IVIg vs MP trial covariate adjustment led to a larger *β* and a smaller p value.

Covariate adjustment increases statistical power, despite the larger standard error.[1, 7] When there are no baseline imbalances, the adjusted conditional estimates will be more extreme than the unadjusted marginal estimates[22]. However, the size and the direction of the difference between the unadjusted and adjusted estimates are dependent on the strength of the prognostic factors and the imbalance in baseline risk between the treatment- and control group in the specific trial and this is shown in our study. When investigating the effectiveness of a medical intervention in rare and heterogeneous neurological diseases, such as GBS, one has to deal with small sample sizes. We therefore recommend performing covariate adjustment in future trials in rare and heterogeneous neurological diseases. For GBS this covariate adjustment should be applied with known predictors for (functional) outcome, specifically age, preceding diarrhea, GBS disability score and MRC sum score[19, 20].

The outcome ‘minimal one grade improvement’ implicitly involves a form of covariate adjustment. The baseline disease severity of the patient is taken into account in the analysis by estimating improvement for each patient from his or her own starting position at admission (Table 4). This principle of a measure of change between baseline and follow up seems attractive to control for baseline imbalance. However, analyzing change does not control for baseline imbalance because of regression to the mean[23, 24]; baseline values are negatively correlated with change because patients with high scores at baseline generally improve more than those with low scores[25]. Therefore covariate adjustment with the absolute baseline value is still preferable over implicitly taking into account baseline severity in the outcome measure ‘improvement’. Moreover, disease severity at baseline is not the only covariate we could adjust for. Especially, the age of the patient will be an important covariate in most neurological diseases.

**Table 4:** Characteristics of four methods of treatment effect analysis in GBS trials. Approach in bold is the recommended approach.

	Takes into account baseline imbalance	Takes into account ordinal nature of the outcome measure
Unadjusted binary logistic regression on cutoff for GBS disability score	NO	NO
Adjusted binary logistic regression on cutoff for GBS disability score	YES	NO
Unadjusted binary logistic regression on ≥ 1 grade improvement on GBS disability score	PARTLY*	NO
Adjusted binary logistic regression on ≥ 1 grade improvement on GBS disability score	YES	NO
Unadjusted proportional odds logistic regression on GBS disability score	NO	YES
**Adjusted proportional odds logistic regression** **on GBS disability score**	**YES**	**YES**
Unadjusted proportional odds logistic regression on Δ GBS disability score	PARTLY*	YES
Adjusted proportional odds logistic regression on Δ GBS disability score	YES	YES

*Only baseline GBS disability score, no other covariates.

Thus, in general, ignoring baseline imbalance between treatment arms in trials may cause invalid conclusions on both the magnitude and significance of the treatment effect estimate compared to analysis using covariate adjustment. The impact on interpretability of treatment effect estimates can be substantial and can be different in several study settings. When designing a trial, the analysis plan should be precisely pre-specified. Also, the covariates that will be used for adjustment should be pre-specified. Previous studies have shown that the stronger the relation of the covariates with outcome, the larger the increase in statistical power with covariate adjustment will be[5, 26, 27]. In GBS, predictors of outcome are relatively well known[19, 20] and therefore pre-specifying important baseline variables for covariate adjustment is possible in GBS trials.

### Proportional odds analysis

It is evident that the GBS disability scale is not a linear scale. For example, improvement from “needs ventilation for at least a part of the day” to “bedridden or chair bound” is not the same improvement as the improvement from “able to walk over 10m open space with help” to “able to walk 10m unassisted but not able to run”. However, whether or not the ordinal outcome under study is a linear scale is not relevant for the validity of the proportional odds analysis. Proportional odds analysis merely requires ordering of outcomes. The proportional odds analysis estimates the treatment effect on each cut-off of the scale, instead of estimating the treatment effect on the difference between the averages scores in the treatment arms, as linear regression. The proportional odds model results in a common OR, which is interpretable as a pooled OR over all ORs for the different cut-offs. The common OR is formally valid if the ORs for each cut-off are the same (the proportional odds assumption). We can, however, interpret the common OR as a summary measure of the treatment effect, even if the ORs differs slightly per cut-off[12, 28]. The common OR can also be interpreted as the average shift over the total ordinal outcome scale caused by the treatment under study[10–13]. Moreover, simulation studies have shown that ordinal analysis is more efficient than binary analysis, even if the proportional odds assumption is violated[11]. Because the ordinal analysis uses the full ordinal outcome scale instead of one dichotomy, the variability will be smaller compared to binary analysis. This was confirmed in our study, where the proportional odds resulted in lower standard errors compared to the binary approaches. Although the importance of applying proportional odds analysis already has been assessed in other diseases, it is still relevant to study this for specific cases like GBS. For example it is important to have more insight in the effect of treatment on the different cut-offs for the specific ordinal outcome measure, in this case the GBS disability score, and see if the proportional odds assumption holds.

In the PE vs IVIg trial, the ORs for each cut-off were very similar and as a result the common OR was also similar. Thus, with the smaller SE, the p value was lower. In contrast, in the IVIg vs IVIg+MP trial, the ORs were more scattered. One explanation is chance: the ORs for the different cut-offs are uncertain, especially at the tails of the outcome scale where numbers are usually small. However, almost all binary ORs have confidence intervals that overlap. Another explanation is that the effect is truly different for different cut-offs, although this is clinically unlikely. The cut-off chosen in the reference approach in the analysis of improvement appeared to be the most optimal cut-off from a statistical perspective, since it was the only cut-off resulting in a significant treatment effect.

However, if we assume a relatively constant treatment effect across the different cut-offs of an ordinal outcome scale, it is unpredictable which cut-off will show the strongest effect. Therefore, the ordinal analysis is a ‘safe’ choice and the common OR is a fair representation of the effect of treatment on the ordinal outcome compared to the binary approach, because it takes into account improvement over all levels of the GBS disability score. Since it is also more efficient, we recommend the use of the full ordinal outcome scale in future trials in rare and heterogeneous neurological diseases. In observational studies, ordinal analyses could be combined with propensity score methods to maximize statistical power.

### Limitations

Patients with missing covariate data were excluded from the analyses. Data from 367 patients were analyzed rather than 372 patients in the original analyses. We did not assess heterogeneous treatment effects according to baseline risk, which could influence the ability of covariate adjustment to improve the statistical power in an RCT. In this study we only investigated GBS which may not fully be representative for other neurological disorders, although covariate adjustment and proportional odds analysis have shown advantages in other fields, such as stroke and traumatic brain injury[3, 4, 7, 12].

### Conclusion and implications

Covariate adjustment corrects for baseline imbalance and increases power. Proportional odds analysis optimally exploits the ordinal nature of outcome scales. A combined approach is advised for reliable and efficient estimation of treatment effects in small RCTs in rare and heterogeneous diseases like GBS.

## Supporting information

S1 Fig Distribution of the GBS disability score at four weeks and improvement on the GBS disability score after four weeks in the IVIg + placebo vs IVIg + Methylprednisolon (IVIg vs MP) trial (a and c) and PE vs IVIg trial (b and d).
(PPTX)

S1 Appendix Overview of a selection of methodological studies considering covariate adjustment and ordinal analysis in RCTs. (DOCX)

## Abbreviations

GBS: Guillain-Barré syndrome
RCT: Randomized Controlled Trial
OR: Odds Ratio
SE: Standard Error
PO: Proportional Odds
RCT: Randomized Controlled Trial
