# Supplementary figures and images for: Efficient design and analysis of randomized controlled trials in rare neurological diseases: An example in Guillain-Barré syndrome
Source: PLoS One. 2019 Feb 20;14(2):e0211404. doi: 10.1371/journal.pone.0211404 (PMC6382155; doi:10.1371/journal.pone.0211404)

## Slide 1
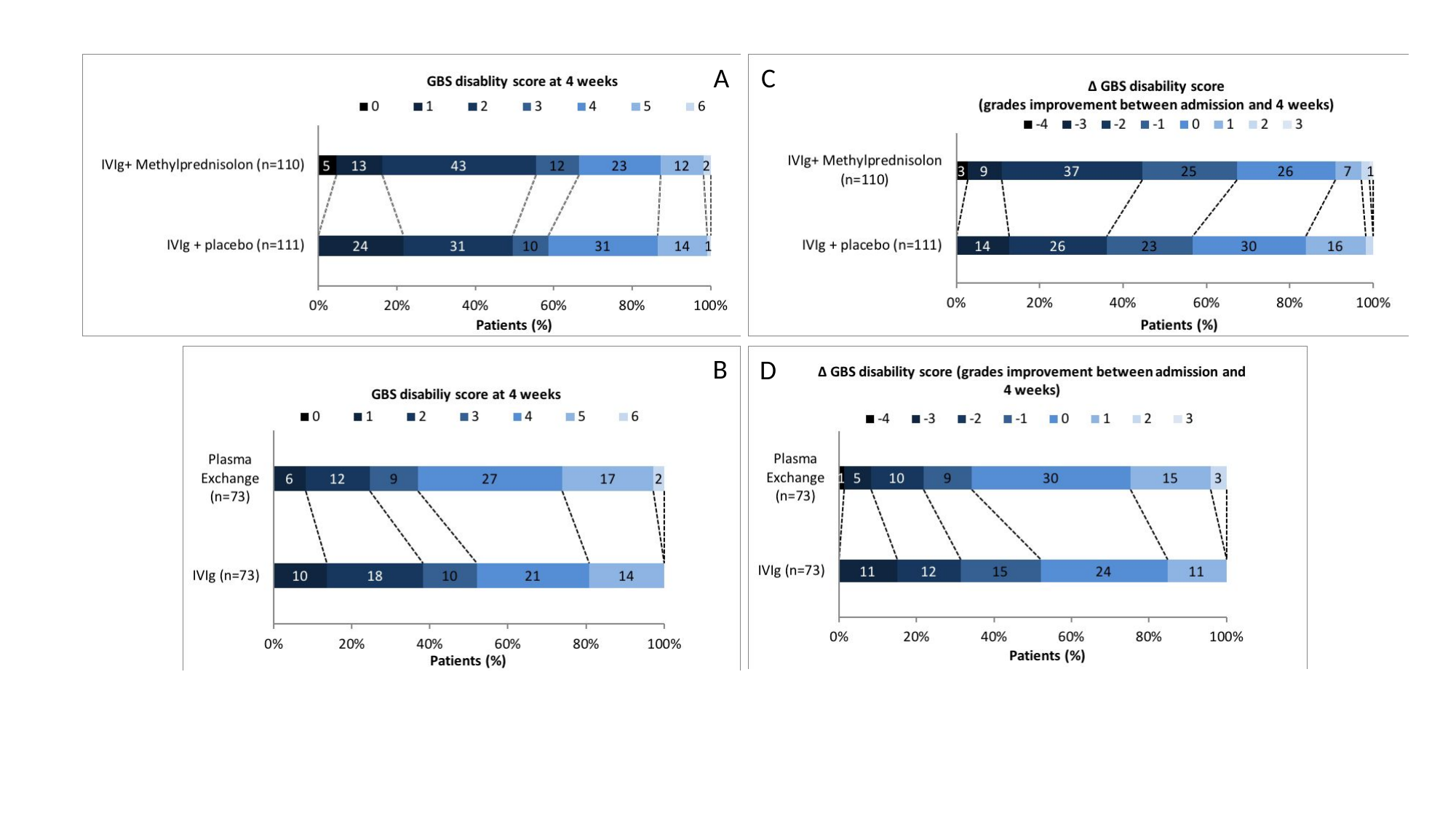

A
C
B
D

Supplement: S1 Fig — Distribution of the GBS disability score at four weeks and improvement on the GBS disability score after four weeks in the IVIg + placebo vs IVIg + Methylprednisolon (IVIg vs MP) trial (a and c) and PE vs IVIg trial (b and d). (PPTX) [file pone.0211404.s001.pptx]
