# Supplementary material for: Efficient design and analysis of randomized controlled trials in rare neurological diseases: An example in Guillain-Barré syndrome
Source: PLoS One. 2019 Feb 20;14(2):e0211404. doi: 10.1371/journal.pone.0211404 (PMC6382155; doi:10.1371/journal.pone.0211404)
Supplement: S1 Appendix — (DOCX) [file pone.0211404.s002.docx]

| First author | Year | Field | Key findings and conclusions |
| --- | --- | --- | --- |
| *Covariate adjustment* | | |  |
| Robinson[[21](#_ENREF_21)] | 1991 | - | In classic linear regression, the adjustment for a non-confounding predictive covariate, results in improved precision, whereas such adjustment in logistic regression results in a loss of precision. However, when testing for a treatment effect in randomized studies, it is always more efficient to adjust for predictive covariates when logistic models are used, and thus in this regard the behavior of logistic regression is the same as that of a classic linear regression. |
| Hauck[[22](#_ENREF_22)] | 1991 | - | In the epidemiologic literature, one finds three criteria for confounding, which we will call the classical (marginal), operational (change-in-estimate) and conditional criteria. We define mavericks to be covariates that satisfy the operational criterion, but not the classical criterion. We present what is known about the problems of mavericks for estimating odds ratios and clarify the interpretation of oddsratios. Key results are: (1) omitting mavericks biases odds ratios towards 1; (2) omitting mavericks cannot artificially introduce an effect in contrast to omitting classical confounders; (3) the operational criterion for confounding corresponds to the conditional criterion when estimating odds ratios, but for relative risks, there are no mavericks (i.e. the classical and operational criterion correspond); and (4) the interpretation of odds ratios obtained from standard methods is that of comparing proportions, not of individual risk. |
| Pocock[[27](#_ENREF_27)] | 2002 | - | When reporting the trial's findings baseline data can be used for i.a. covariate-adjusted analyses which aim to refine the analysis of the overall treatment difference by taking account of the fact that some baseline characteristics are related to outcome and may be unbalanced between treatment groups and baseline comparisons which compare the baseline characteristics of patients in each treatment group for any possible differences. This paper examines how these issues are currently tackled in the medical journals, based on a recent survey of 50 trialreports in four major journals. Key issues include: inconsistencies in the use of covariate-adjustment; the lack of clear guidelines on covariate selection; the overuse of baseline comparisons in some studies; the misuses of significance tests for baseline comparability, and the need for trials to have a predefined statistical analysis plan for all these uses of baseline data. |
| Hernandez[[6](#_ENREF_6)] | 2004 | - | Logistic regression analysis was applied to simulated data sets (n=360) with different treatment effects, covariate effects, outcome incidences, and covariate prevalences. Treatment effects were estimated with or without adjustment for a single dichotomous covariate. The power was highest with prespecified adjustment. The potential reduction in sample size was higher with stronger covariate effects (from 3 to 46%, at 50% outcome incidence and covariate prevalence) and independent of the treatment effect. At lower outcome incidences and/or covariate prevalences, the reduction was lower. |
| Hernandez[[29](#_ENREF_29)] | 2005 | Traumatic brain injury | 18 RCTs (n = 6439) were identified in a systematic review of therapeutic phase III RCTs, including adult patients with acute, moderate-to-severe TBI to assess actual reporting of covariate adjustment according to the Consolidated Standards of Reporting Trials (CONSORT) recommendations. Five RCTs reported covariate adjustment. The number of covariates was limited (<=5), most frequently including age. Many covariates were outcome predictors. Four RCTs reported only adjusted treatment effects as the main efficacy parameter. The reported covariate adjustment in TBI trials had several methodological shortcomings. Appropriate performance and reporting of covariate adjustment and subgroup analysis should be considerably improved in future TBI trials because interpretation of treatment benefits may be misleading otherwise. |
| Hernandez[[5](#_ENREF_5)] | 2006 | Traumatic brain injury | Individual patient data from seven therapeutic phase III randomized clinical trials (RCTs; n = 6166) in moderate or severe TBI, and three TBI surveys (n = 2238) were used to calculate the potential sample size reduction obtained by adjustment of a hypothetical treatment effect for one to seven predictors with logistic regression models. The distribution of predictors was more heterogeneous in surveys than in trials. Adjustment of the treatment effect for the strongest predictors (age, motor score, and pupillary reactivity) yielded a reduction in sample size of 16-23% in RCTs and 28-35% in surveys. Adjustment for seven predictors yielded a reduction of about 25% in most studies: 20-28% in RCTs and 32-39% in surveys. |
| Optimizing the Analysis of Stroke Trials (OAST) Collaboration[[4](#_ENREF_4)] | 2009 | Acute stroke | Data from 23 stroke trials (N = 25 674) assessing functional outcome were included. Unadjusted and adjusted ordinal logistic regression models were compared using simulated data from each trial (10 000 simulations per trial). Three levels of treatment effect were assessed with ORs of 0.95, 0.74, and 0.57. Adjusting for prognostic factors in stroke trials can reduce sample size by at least 20% to 30% (the lower interquartile range) for a given power and is similar across all 3 treatment effects |
| Roozenbeek[[7](#_ENREF_7)] | 2009 | Traumatic brain injury | Statistical modeling studies in three surveys and six randomized controlled trials were performed using individual patient data from the IMPACT database. Covariate adjustment reduced sample sizes by 30% in surveys and 16% in RCTs. Covariate adjusted analysis in a broadly selected group of patients is advisable if a uniform treatment effect is assumed, since there is no decrease in recruitment. |
| Steyerberg[[8](#_ENREF_8)] | 2010 | Acute myocardial infarction | The effects of adjustment (correction for imbalance and stratification) were studied with logistic regression analysis in the GUSTO-I trial. When adjusted for 17 characteristics, the odds ratio was 0.820, an increase of 25% compared to the unadjusted odds ratio. The increase in effect estimate was largely explained by the stratification effect and only partly by imbalance of predictors. Adjustment for predictive baseline characteristics, even when largely balanced, may lead to clearly different estimates of the treatment effect on mortality rates. |
| Ciolino[[30](#_ENREF_30)] | 2011 | Acute ischemic stroke | Based on data from a clinical trial of acute ischemic stroke treatment, computer simulations were used to create scenarios varying from the best possible baseline covariate balance to the worst possible imbalance, with multiple balance levels between the two extremes. Our simulation results show that the worst possible imbalance is highly unlikely, but it can still occur under simple random allocation. Also, power loss could be nontrivial if balancing distributions of important continuous covariates were ignored even if adjustment is made in the analysis for important covariates. This situation, although unlikely, is more serious for trials with a small sample size and for covariates with large influence on primary outcome. |
| Turner[[9](#_ENREF_9)] | 2012 | Traumatic brain injury | 14-day mortality was analyzed in 9,497 TBI patients in the CRASH trial of corticosteroid vs. placebo. Adjustment was made using logistic regression for baseline covariates of two validated risk models (IMPACT and CRASH) derived from external data. The relative sample size (RESS) measure, defined as the ratio of the sample size required by an adjusted analysis to attain the same power as the unadjusted reference analysis, was used to assess the impact of adjustment. RESS of 0.79 and 0.73 were obtained by adjustment using the IMPACT and CRASH models, respectively, which, for example, implies an increase from 80% to 88% and 91% power, respectively. |
| Ciolino[[31](#_ENREF_31)] | 2013 | Acute stroke | This article uses simulation to quantify the benefit of covariate adjustment in logistic regression. Results suggest that if adjustment is not possible or unplanned in a logistic setting, balance in continuous covariates can alleviate some (but never all) of the shortcomings of unadjusted analyses. |
| Garofolo[[32](#_ENREF_32)] | 2013 | Acute stroke | Using a current stroke clinical trial and its pilot studies to guide simulation parameters, 1,000 clinical trials were simulated at varying sample sizes under several treatment effects to assess power and type I error. Covariate-adjusted and unadjusted logistic regressions were used to estimate the treatment effect under each scenario. Under various treatment effect settings, the operating characteristics of the unadjusted and adjusted analyses do not substantially differ. Power and type I error are preserved for both the unadjusted and adjusted analyses. |
| Thompson[[1](#_ENREF_1)] | 2015 | Stroke and acute myocardial infarction | In two large trial data sets GUSTO-I (N = 30,510) and IST (N = 18,372) random samples (500,000 times) of sizes 300 and 5,000 per arm were repeatedly drawn, and simulated each primary outcome using the control arms. The power gained from a covariate adjusted analysis for small and large samples was between 5% and 6%. Similar proportions of discordance with respect to statistical significance were noted irrespective of the sample size in both the GUSTO-I and the IST data sets. |
| *Ordinal outcome analysis* | | |  |
| Valenta[[10](#_ENREF_10)] | 2006 | - | In this article conceptual and methodological aspects of employing proportional odds logistic regression for a three level ordinal factor as a suitable alternative to ordinary logistic regression when dealing with limited uncertainty in classifying clinical outcome as a binary variable are reviewed. Classifying a measurable clinical outcome as a dichotomous variable often involves difficulty with borderline cases that could fairly be assigned either of the two binary class memberships. In such situations the indicated class membership is often highly subjective and subject to, for instance, a measurement error. In other situations the intermediate level of a three-level ordinal factor may sometimes be explicitly reserved for cases which could likely belong to either of the two binary classes. |
| Optimizing the Analysis of Stroke Trials (OAST) Collaboration[[3](#_ENREF_3)] | 2007 | Acute stroke | Data from 55 datasets (47 trials, 54,173 patients) from acute, rehabilitation and stroke unit trials studying the effects of interventions were used to asses which statistical approaches are most efficient in analyzing outcomes from stroke trials. The test results differed substantially so that approaches which use the ordered nature of functional outcome data (ordinal logistic regression, t test, robust ranks test, bootstrapping the difference in mean rank) were more efficient statistically than those which collapse the data into 2 groups (chi(2); ANOVA, P<0.001). The findings were consistent across different types and sizes of trial and for the different measures of functional outcome. |
| Saver[[13](#_ENREF_13)] | 2007 | Acute stroke | Dichotomized, global statistic, responder, and shift analyses each offer distinctive benefits and drawbacks. Choice of primary end point analytic technique should be tailored to the study population, expected treatment response, and study purpose. Shift analysis generally provides the most comprehensive index of a treatment's clinical impact. Shift analysis gauges change in outcome distributions over the full range of ascertained outcomes, incorporating benefit and harm at all health state transitions valued by patients and clinicians, and often increasing study power. |
| Senn[[33](#_ENREF_33)] | 2009 | - | Biostatisticians have frequently uncritically accepted the measurements provided by their medical colleagues engaged in clinical research, which often involve considerable loss of information. Particularly, unfortunate is the widespread use of the so-called 'responder analysis', which may involve not only a loss of information through dichotomization, but also extravagant and unjustified causal inference regarding individual treatment effects at the patient level, and, increasingly, the use of the so-called number needed to treat scale of measurement. Other problems involve inefficient use of baseline measurements, the use of covariates measured after the start of treatment, the interpretation of titrations and composite response measures. Statisticians should pay more attention to this aspect of their work. |
| McHugh[[11](#_ENREF_11)] | 2010 | Traumatic brain injury | This study was based on simulations, which were built around a database of patient-level data extracted from eight Phase III trials and three observational studies in traumatic brain injury. Two different putative treatment effects were explored, one which followed the proportional odds model, and the other which assumed that the effect of the intervention was to reduce the risk of death without changing the distribution of outcomes within survivors. The simulation results show substantial efficiency gains. Use of the sliding dichotomy allows sample sizes to be reduced by up to 40% without loss of statistical power. The proportional odds model gives modest additional gains over and above the gains achieved by use of the sliding dichotomy. |
| Roozenbeek[[12](#_ENREF_12)] | 2011 | Traumatic brain injury | Two techniques for ordinal analysis were applied using data from the CRASH trial (n = 9,554): proportional odds analysis and the sliding dichotomy approach, where the GOS is dichotomized at different cut-offs according to baseline prognostic risk. These approaches were compared to dichotomous analysis. Ordinal analysis with proportional odds regression or sliding dichotomy showed highly statistically significant treatment effects, with 2.05-fold and 2.56-fold higher information density compared to the dichotomous approach respectively. Analysis of the CRASH trial data confirmed that ordinal analysis of outcome substantially increases statistical power. |
| Diaz[[34](#_ENREF_34)] | 2016 | Acute stroke | A general method for estimating the effect of a treatment on an ordinal outcome in randomized trials is presented. The method is robust in that it does not rely on the proportional odds assumption. Our estimator leverages information in prognostic baseline variables, and has all of the following properties: (i) it is consistent; (ii) it is locally efficient; (iii) it is guaranteed to have equal or better asymptotic precision than both the inverse probability-weighted and the unadjusted estimators. The estimator is demonstrated in simulations based on resampling from a completed randomized clinical trial of a new treatment for stroke; we show potential gains of up to 39% in relative efficiency compared to the unadjusted estimator. |

Additional references:

29. Hernandez AV, Steyerberg EW, Taylor GS, Marmarou A, Habbema JD, Maas AI. Subgroup analysis and covariate adjustment in randomized clinical trials of traumatic brain injury: a systematic review. Neurosurgery. 2005;57(6):1244-53; discussion -53.

30. Ciolino J, Zhao W, Martin R, Palesch Y. Quantifying the cost in power of ignoring continuous covariate imbalances in clinical trial randomization. Contemp Clin Trials. 2011;32(2):250-9.

31. Ciolino JD, Martin RH, Zhao W, Jauch EC, Hill MD, Palesch YY. Covariate imbalance and adjustment for logistic regression analysis of clinical trial data. J Biopharm Stat. 2013;23(6):1383-402.

32. Garofolo KM, Yeatts SD, Ramakrishnan V, Jauch EC, Johnston KC, Durkalski VL. The effect of covariate adjustment for baseline severity in acute stroke clinical trials with responder analysis outcomes. Trials. 2013;14:98.

33. Senn S, Julious S. Measurement in clinical trials: a neglected issue for statisticians? Stat Med. 2009;28(26):3189-209.

34. Diaz I, Colantuoni E, Rosenblum M. Enhanced precision in the analysis of randomized trials with ordinal outcomes. Biometrics. 2016;72(2):422-31.
